# Network-Based Methods for Prediction of Drug-Target Interactions

**DOI:** 10.3389/fphar.2018.01134

**Published:** 2018-10-09

**Authors:** Zengrui Wu, Weihua Li, Guixia Liu, Yun Tang

**Affiliations:** Shanghai Key Laboratory of New Drug Design, School of Pharmacy, East China University of Science and Technology, Shanghai, China

**Keywords:** drug-target interaction, network-based method, target prediction, systems pharmacology, systems toxicology, drug repurposing

## Abstract

Drug-target interaction (DTI) is the basis of drug discovery. However, it is time-consuming and costly to determine DTIs experimentally. Over the past decade, various computational methods were proposed to predict potential DTIs with high efficiency and low costs. These methods can be roughly divided into several categories, such as molecular docking-based, pharmacophore-based, similarity-based, machine learning-based, and network-based methods. Among them, network-based methods, which do not rely on three-dimensional structures of targets and negative samples, have shown great advantages over the others. In this article, we focused on network-based methods for DTI prediction, in particular our network-based inference (NBI) methods that were derived from recommendation algorithms. We first introduced the methodologies and evaluation of network-based methods, and then the emphasis was put on their applications in a wide range of fields, including target prediction and elucidation of molecular mechanisms of therapeutic effects or safety problems. Finally, limitations and perspectives of network-based methods were discussed. In a word, network-based methods provide alternative tools for studies in drug repurposing, new drug discovery, systems pharmacology and systems toxicology.

## Introduction

As the rapid development of systems biology and network pharmacology, the drug discovery paradigm has changed from the linear mode “one drug → one target → one disease" to the network mode “multi-drugs → multi-targets → multi-diseases" (Hopkins, 2008; Medina-Franco et al., 2013; Anighoro et al., 2014). The new paradigm means that a single drug might act on multiple targets *in vivo*, rather than selectively bind to one target, which is more consistent with those observed in reality (Roth et al., 2004; Paolini et al., 2006; Yildirim et al., 2007). For a drug, its polypharmacological profile (i.e., on-target and off-target effects) could lead to both desired therapeutic effects and undesired safety problems (Roth et al., 2004; Keiser et al., 2009; Besnard et al., 2012; Lounkine et al., 2012; Anighoro et al., 2014; Zhang et al., 2015). Hence, systematic identification of drug-target interactions (DTIs) is essential in drug discovery, which could help maximize therapeutic effects while minimizing safety problems.

The traditional way to identify DTIs is via biological experiments, for example, to determine the inhibition constant (K_i_), dissociation constant (K_d_), half-maximal inhibitory concentration (IC_50_) or half-maximal effective concentration (EC_50_) values between drugs (e.g., approved drugs, drug candidates in clinical trials, drugs withdrawn from the market and drug-like new chemical entities) and target proteins by *in vitro* or *in vivo* assays. However, it is time-consuming and costly to determine all possible DTIs experimentally and systematically. Over the past decade, the development of various computational methods has provided valuable strategies for the systematic prediction of potential DTIs due to their high efficiency and low costs (Zheng et al., 2013; Chen et al., 2016; Lavecchia and Cerchia, 2016). On one hand, according to the type of prediction results, these methods can be divided into two categories, namely qualitative (i.e., classification) and quantitative (i.e., regression) methods. On the other hand, these methods can be roughly divided into several categories, including molecular docking-based, pharmacophore-based, similarity-based, machine learning-based, and network-based methods, although their concepts could overlap each other.

Molecular docking-based methods are traditional approaches based on the three-dimensional (3D) structures of targets, which have been widely used in DTI prediction (Rognan, 2010; Waszkowycz et al., 2011; Ma et al., 2013). These methods use scoring functions to evaluate DTIs, which can provide quantitative docking scores correlated with binding affinities (Li et al., 2014; Liu et al., 2017). For one or a few given targets such as estrogen receptors (Shen et al., 2010) or HIV-1 integrase (Hu et al., 2012b), potential active compounds can be prioritized by molecular docking. Reversely, for a given drug or new chemical entity, reverse docking (also known as inverse docking) can be used to predict potential targets for it (Chen and Zhi, 2001; Tang et al., 2006; Rognan, 2010). Several web applications, such as TarFisDock (Li et al., 2006) and DRAR-CPI (Luo et al., 2011), were built for docking-based target fishing.

Pharmacophore-based methods can be further divided into two subtypes, namely structure-based and ligand-based pharmacophore mapping. Both of them can be used in DTI prediction (Rognan, 2010; Yang, 2010). For example, PharmMapper is a web server which can predict potential targets for a submitted compound via structure-based pharmacophore mapping (Liu et al., 2010). In practical application, to improve the predictive accuracy, a strategy combining pharmacophore mapping and molecular docking is often used to predict potential DTIs, such as to find new ligands for a given receptor (Chen et al., 2014).

Similarity searching is also a traditional approach for DTI prediction (Willett et al., 1998), based on a hypothesis that similar drugs share similar targets and vice versa. Various types of similarity such as two-dimensional (2D) fingerprint-based similarity (Willett, 2006), 3D shape similarity (Hu et al., 2012a), and phenotypic similarity (Campillos et al., 2008) can be employed in similarity-based methods. For example, two web applications named similarity ensemble approach (SEA) (Keiser et al., 2007) and ChemMapper (Gong et al., 2013) use 2D and 3D similarity, respectively, to predict potential DTIs.

Machine learning is a general approach used in DTI prediction, which has been developing rapidly in these years (Ding et al., 2014; Chen et al., 2016). For example, we proposed two machine learning-based methods, namely multitarget-QSAR (mt-QSAR) and computational chemogenomic methods (Cheng et al., 2012c). Based on mt-QSAR, a web application named CPI-Predictor was developed for free use (Cheng et al., 2012c). Besides traditional machine learning techniques, deep learning techniques have been applied in DTI prediction recently (Tian et al., 2016; Wen et al., 2017). Currently, although 3D structure data of targets can be used in building machine learning models (Hwang et al., 2017), molecular descriptors and protein sequence descriptors are much more commonly used (Cheng et al., 2012c; Yu et al., 2012; Ding et al., 2014).

The above-mentioned methods have shown high accuracies and robustness in DTI prediction (Zheng et al., 2013; Chen et al., 2016; Lavecchia and Cerchia, 2016). However, there are still several pitfalls among them. Structure-based methods such as molecular docking and structure-based pharmacophore mapping rely on 3D structures of targets (Tang et al., 2006; Rognan, 2010). Hence, they are often limited by lack of high-quality 3D structures. For example, G protein-coupled receptors (GPCRs) are the largest protein family consisting of more than 800 members (Stevens et al., 2013), but only approximately 30 of them have resolved crystal structures yet (Xiang et al., 2016). Ligand-based pharmacophore mapping relies on the proper selection of training set compounds for building pharmacophore models, a problem often confused by users, even experienced ones (Yang, 2010). Similarity-based methods rely on similarity and also limited by the similarity. For instance, the methods based on chemical structure similarity could be difficult to find active compounds with novel scaffolds. The methods based on phenotypic similarity could be limited by lack of enough phenotypic data. In general, building machine learning models (especially supervised learning models) for DTI prediction requires both positive samples and negative samples (i.e., active and inactive DTIs validated by experiments) (Cheng et al., 2012c; Yu et al., 2012; Ding et al., 2014; Chen et al., 2016). However, it is always difficult to find enough number of experimentally validated negative samples with gold standard from publicly available database and literature (Cheng et al., 2012c; Yu et al., 2012). Although strategies such as “one versus the rest” (Cheng et al., 2012c) can be used to generate enough negative samples, the model performance is often influenced by the low-quality negative samples.

Compared to these methods, network-based methods have demonstrated great advantages. At first, network-based methods do not rely on 3D structures of targets or negative samples. These methods are derived from recommendation algorithms used in recommender systems (Lu et al., 2012) and link prediction algorithms in complex networks (Lu and Zhou, 2011). For example, more than ten years ago, Zhou et al. (2007) proposed a recommendation algorithm named network-based inference (NBI), also known as probabilistic spreading (ProbS) (Zhou et al., 2010), to recommend possible future likes (called objects) for users based on the known preference data of the users. By treating drugs and targets as users and objects, respectively, this algorithm was grafted into the research area of DTI prediction (Cheng et al., 2012b). As one of the simplest network-based methods, NBI can predict potential DTIs only using the known DTI network (i.e., positive samples), without any additional information such as chemical structures, protein structures or sequences. In the next few years, several new network-based methods were developed based on NBI (Cheng et al., 2012d; Wu et al., 2016, 2017) and other recommendation algorithms or link prediction algorithms (Cheng et al., 2013c; Duran et al., 2017). These methods all have the advantages that are independence on 3D structures of targets and negative samples, which enable them to cover much larger target space. Secondly, network-based methods are simple and fast. These methods predict potential DTIs only by performing simple physical processes such as resource diffusion (Cheng et al., 2012b,d; Wu et al., 2016, 2017), collaborative filtering (Cheng et al., 2012b, 2013c), and random walk (Chen et al., 2012) on networks. Considering networks can be represented by matrices, these processes can be described by simple matrix operations such as matrix multiplication mathematically (Chen et al., 2012; Cheng et al., 2012b; Wu et al., 2017). Compared with some complex structure-based and machine learning-based methods, the calculation procedures are simple and can be parallelized easily. Hence, network-based methods often run fast on computers.

In this article, we focused on network-based methods for DTI prediction. We first introduced the data sources for network construction and the methodologies of several representative network-based methods, especially a series of methods developed on the basis of NBI (we called them “NBI series methods”) (Cheng et al., 2012b,d; Wu et al., 2016, 2017). Subsequently, the evaluation approaches and indicators were introduced briefly. Then, the emphasis was put on their applications in a wide range of fields, including target prediction and elucidation of molecular mechanisms of therapeutic effects or safety problems. These applications suggest that network-based methods provide alternative tools for studies in drug repurposing, new drug discovery, systems pharmacology and systems toxicology.

## Data Sources For Network Construction

Networks, especially DTI networks, are one of the most important bases of network-based models. To construct reliable networks, it is necessary to have sufficient amount of high-quality data. Fortunately, we are living in an era of big data (Schadt, 2012; Ma’ayan et al., 2014). There are a large number of data available freely online, from small molecules to biomacromolecules, from structures to properties, from raw data to organized data in different topics (Chen et al., 2016). Herein, we introduced several well-known data sources for network construction.

There are different ways to construct DTI networks. For example, we can download prepared DTI data from public databases such as DrugBank (Wishart et al., 2018) and Therapeutic Target Database (Li et al., 2018). The downloaded DTI pairs can be used to construct DTI networks directly. However, DTI data from these databases are not quantitative, because the experimentally determined activity values of the DTIs are not provided. It may cause problems in merging DTI data from different sources. By contrast, as shown in **Table 1**, there are many databases can provide experimentally determined DTI data with quantitative activity values such as K_i_, K_d_, IC_50_, and EC_50_ values, including BindingDB (Gilson et al., 2016), Binding MOAD (Ahmed et al., 2015), ChEMBL (Gaulton et al., 2017), DrugCentral (Ursu et al., 2017), IUPHAR/BPS Guide to PHARMACOLOGY (Harding et al., 2018), PDBbind-CN (Liu et al., 2015), PDSP K_i_ Database (Roth et al., 2000), PubChem BioAssay (Wang et al., 2017), RCSB Protein Data Bank (Rose et al., 2017), SuperTarget (Hecker et al., 2012), STITCH (Szklarczyk et al., 2016), TDR Targets (Magarinos et al., 2012), Thomson Reuters Integrity, etc. After collecting quantitative DTI data from these databases, we can use the same criteria for data filtering and merging. Then, one or more DTI networks can be constructed based on the prepared DTI pairs as below. If a drug and a target are validated to interact with each other by experiments (e.g., K_i_, K_d_, IC_50_ or EC_50_ ≤ 10 μM), the node representing the drug and the node representing the target are linked by an edge.

**Table 1:** Several representative databases containing experimentally determined DTI data with quantitative activity values.

Name	Free use	Website	Reference
BindingDB	√	http://www.bindingdb.org/	Gilson et al., 2016
Binding MOAD	√	http://www.bindingmoad.org/	Ahmed et al., 2015
ChEMBL	√	http://www.ebi.ac.uk/chembl/	Gaulton et al., 2017
DrugCentral	√	http://drugcentral.org/	Ursu et al., 2017
IUPHAR/BPS Guide to PHARMACOLOGY	√	http://www.guidetopharmacology.org/	Harding et al., 2018
PDBbind-CN	√	http://www.pdbbind-cn.org/	Liu et al., 2015
PDSP K_i_ Database	√	http://pdsp.unc.edu/databases/kidb.php	Roth et al., 2000
PubChem BioAssay	√	http://www.ncbi.nlm.nih.gov/pcassay/	Wang et al., 2017
RCSB Protein Data Bank	√	http://www.rcsb.org/	Rose et al., 2017
SuperTarget	√	http://insilico.charite.de/supertarget/	Hecker et al., 2012
STITCH	√	http://stitch.embl.de/	Szklarczyk et al., 2016
TDR Targets	√	http://tdrtargets.org/	Magarinos et al., 2012
Thomson Reuters Integrity	×	http://integrity.thomson-pharma.com/	

In addition to known DTIs, we can also use other types of data to aid the DTI prediction. For example, chemical substructures can be generated for drugs using chemoinformatics software such as Open Babel (O’Boyle et al., 2011) and PaDEL-Descriptor (Yap, 2011). Anatomical Therapeutic Chemical classification (ATC) codes of drugs can be obtained from databases such as DrugBank (Wishart et al., 2018), DrugCentral (Ursu et al., 2017), and KEGG DRUG (Kanehisa et al., 2017). Side effects of drugs can be collected from Comparative Toxicogenomics Database (CTD) (Davis et al., 2017), SIDER (Kuhn et al., 2016), and OFFSIDES (Tatonetti et al., 2012). Sequences of target proteins can be downloaded from UniProt knowledgebase (Bateman et al., 2017). Using these data, we can generate more types of data. For example, chemical similarity of drug-drug pairs can be calculated using their substructures (Chen et al., 2012; Cheng et al., 2012b). Therapeutic and side-effect similarity networks of drug-drug pairs can be calculated using their ATC codes and side effects, respectively (Cheng et al., 2013c). Protein sequence similarity of target-target pairs can be calculated using their sequences (Chen et al., 2012; Cheng et al., 2012b). Using these different similarity data, various similarity networks can be constructed, which may be used in network-based methods together with DTI networks.

## Methodologies Of Network-Based Methods

As described in the INTRODUCTION section, network-based methods are derived from recommendation algorithms (Lu et al., 2012) and link prediction algorithms (Lu and Zhou, 2011). Previous reviews have suggested that these methods are different from machine learning-based methods and similarity-based methods (Ding et al., 2014; Chen et al., 2016). Although many recommendation algorithms and link prediction algorithms have been proposed up to date (Clauset et al., 2008; Guimera and Sales-Pardo, 2009; Lu and Zhou, 2011; Lu et al., 2012; Pan et al., 2016), few of them were applied in DTI prediction. In this section, we introduced the methodologies of several representative network-based methods, as listed in **Table 2**.

**Table 2:** Several representative types of network-based methods for DTI prediction.

Type	Name	Website	Reference
NBI series methods	NBI	http://lmmd.ecust.edu.cn/database/dti/	Cheng et al., 2012b
	EWNBI		Cheng et al., 2012d
	NWNBI		Cheng et al., 2012d
	SDTNBI	http://lmmd.ecust.edu.cn/methods/sdtnbi/	Wu et al., 2017
	bSDTNBI	http://lmmd.ecust.edu.cn/methods/bsdtnbi/	Wu et al., 2016
Similarity inference methods	DBSI		Cheng et al., 2012b
	TBSI		Cheng et al., 2012b
	DSESI		Cheng et al., 2013c
	DTSI		Cheng et al., 2013c
Random walk-based methods	NRWRH		Chen et al., 2012
Local-community-paradigm methods	CAR	http://sites.google.com/site/carlovittoriocannistraci/5-datasets-and-matlab-code/bipartite-link-predictors/	Duran et al., 2017
	CJC		Duran et al., 2017
	CPA		Duran et al., 2017
	CAA		Duran et al., 2017
	CRA		Duran et al., 2017
Simple path-based method	DASPfind	http://www.cbrc.kaust.edu.sa/daspfind/	Ba-Alawi et al., 2016

### NBI Series Methods

#### NBI

**FIGURE 1:**
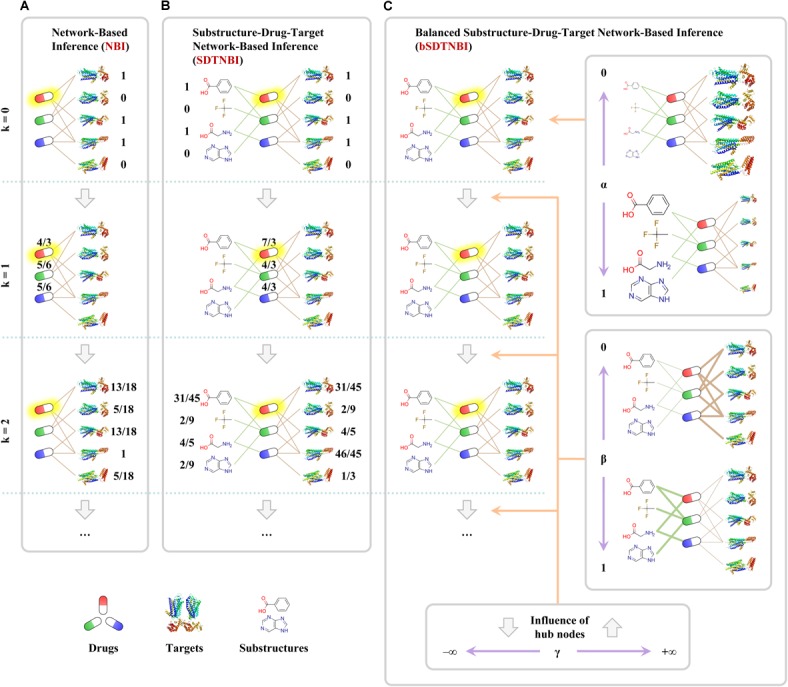
Examples of predicting potential targets for an example drug highlighted with yellow shadow via three network-based methods: **(A)** network-based inference (NBI), **(B)** substructure-drug-target network-based inference (SDTNBI), **(C)** balanced substructure-drug-target network-based inference (bSDTNBI). Drugs, targets and substructures were represented as capsules, ribbons and structural fragments, respectively.

NBI performs resource-diffusion processes on the known DTI network to prioritize potential DTIs (Cheng et al., 2012b). As shown in **Figure 1A**, if we want to predict potential targets for an example drug in the known DTI network (symbolized as *D*_i_), the following steps can be performed. Initially, one unit of resource is allocated to each of the neighbor nodes of *D*_i_ (i.e., the target nodes linked with *D*_i_). Then, in the first resource-diffusion process, each target node equally spreads its resource to its neighbor nodes (i.e., the drug nodes linked with the target node). In the second resource-diffusion process, each drug node equally spreads its resource to its neighbor nodes (i.e., the target nodes linked with the drug node). After the two resource-diffusion processes, for each target in the known DTI network (symbolized as *T*_j_), the amount of the resource located in *T*_j_ can be recognized as the predictive score of the interaction between *D*_i_ and *T*_j_. Higher score means higher probability that *D*_i_ can interact with *T*_j_. Using the same way, we can systematically predict potential DTIs for all drugs in the known DTI network. Although the resource-diffusion processes can be repeated continuously, we usually perform only two resource-diffusion processes.

As one of the simplest network-based methods, NBI only uses a known DTI network as input. Although this characteristic makes NBI run fast, it limits the application domain of NBI enormously. NBI can only predict DTIs for the drugs and targets within the known DTI network. It cannot predict potential targets for new chemical entities without known targets (e.g., newly extracted natural products and newly synthesized compounds) and targets without known ligands (e.g., orphan receptors), owing to the fact that they cannot be interlinked with the known DTI network. Moreover, in the design of NBI, DTIs do not have any attributes, such as interaction types or binding affinities. Hence, it is necessary to further improve the NBI method.

#### EWNBI and NWNBI

To investigate whether introduction of edge or node weights would improve the performance of the original NBI method, we further developed two weighted NBI methods, namely edge-weighted network-based inference (EWNBI) and node-weighted network-based inference (NWNBI) (Cheng et al., 2012d). The two methods use different strategies to improve the predictive accuracy. EWNBI assigns weighted values to all edges (i.e., DTIs) in the known DTI network according to their K_i_ or IC_50_ values, whereas NWNBI introduces a tunable parameter to adjust the influence of hub nodes. The designs are reasonable. However, the performance of EWNBI was marginally worse than NBI. The performance of NWNBI was only marginally better than NBI after parameter optimization. These results suggested that adding node or edge weights directly into the original NBI method is not an appropriate strategy for performance improvement. Basically, EWNBI and NWNBI did not make a breakthrough in the old framework of NBI. All the defects of NBI were not repaired.

#### SDTNBI

In order to overcome one of the aforementioned pitfalls that NBI cannot predict targets for new chemical entities, we proposed a new network-based method entitled substructure-drug-target network-based inference (SDTNBI) (Wu et al., 2017). SDTNBI employs chemical substructures to bridge the gap between known DTI network and the new chemical entities. Specifically, after generating chemical substructures for both drugs in the known DTI network and new chemical entities, the known DTI network and the new chemical entities can be integrated in a structure-drug-target network via linking drugs and new chemical entities by their substructures. As shown in **Figure 1B**, resource-diffusion processes can be performed on the substructure-drug-target network to prioritize possible targets for known drugs and new chemical entities. The prediction steps for an example drug (symbolized as *D*_i_) can be described as below, which are similar to those of NBI. Because new chemical entities can be seen as special drugs without known targets, both drugs in the known DTI network and new chemical entities are called “drugs” to facilitate the description.

Initially, the number of resource-diffusion processes (symbolized as *k*, as shown in **Figure 1**) = 0, one unit of resource is located in each of the neighbor nodes of *D*_i_. Then, in the first resource-diffusion process (*k* = 1), each substructure equally spreads its resource to its neighbor nodes, meanwhile each target node also equally spreads its resource to its neighbor nodes. In the second resource-diffusion process (*k* = 2), each drug node equally spreads its resources to its neighbor nodes. The resource-diffusion processes can be repeated continuously. When the *k* value is an even number, for each target *T*_j_, the amount of the resource located in *T*_j_ can be recognized as the predictive score of the interaction between *D*_i_ and *T*_j_. Using the same way, we can predict potential DTIs for all drugs and new chemical entities systematically and efficiently.

The development of SDTNBI overcame one of the pitfalls of NBI successfully. However, its performance is often worse than NBI (Wu et al., 2017). Considering that the substructure-drug-target network contains different types of nodes and different types of edges, the possible reason for this underperformance is that potential unbalance exists in the resource-diffusion processes of SDTNBI.

#### bSDTNBI

Recently, we made an improvement on SDTNBI by introducing three tunable parameters, namely α, β, and γ, into it, which led to the balanced substructure-drug-target network-based inference (bSDTNBI) (Wu et al., 2016). As shown in **Figure 1C**, the first parameter α ∈ [0,1] is used to adjust which type of nodes will obtain more amount of resource in the initial resource allocation. The second parameter β ∈ [0,1] is used to adjust which type of edges will have larger weighted values in the resource-diffusion processes. The third parameter γ ∈ (-∞,+∞) is used to adjust the influence of hub nodes in the resource-diffusion processes, where γ > 0 and γ < 0 mean strengthen and weaken the influence of hub nodes, respectively. A detailed mathematical description of NBI, SDTNBI, and bSDTNBI was put in the Supplementary data of our previous study (Wu et al., 2018). To compare the performance of bSDTNBI with those of aforementioned NBI and SDTNBI, we performed systematic evaluation and then found that bSDTNBI outperformed SDTNBI and was comparable to NBI when the three parameters were optimized (Wu et al., 2016).

Although aforementioned SDTNBI and bSDTNBI have the ability to prioritize possible targets for various types of compounds, they still cannot predict potential ligands for those targets without known ligands. Moreover, the interaction type and binding affinity of DTIs are not considered yet. Hence, it is still necessary to make further improvements on NBI series methods.

### Similarity Inference Methods

Similarity inference methods are derived from collaborative-filtering algorithms in recommender systems (Huang et al., 2007). These methods have characteristics of both network-based methods and similarity-based methods, which use both topology information of the known DTI network and similarity information to predict potential DTIs (Cheng et al., 2012b, 2013c).

For example, in the previous study of the NBI method (Cheng et al., 2012b), we also proposed two similarity inference methods named drug-based similarity inference (DBSI) and target-based similarity inference (TBSI). DBSI is based on the hypothesis that drugs with similar 2D chemical structures tend to act on similar targets. TBSI is based on the hypothesis that proteins with similar sequences tend to be targeted by similar drugs. However, both DBSI and TBSI underperformed NBI in a systematic evaluation. This underperformance may be caused by the redundancy in the drug similarity and target similarity.

Subsequently, we developed two network-based methods named drug side-effect similarity inference (DSESI) and drug therapeutic similarity inference (DTSI) (Cheng et al., 2013c). DSESI and DTSI are based on the hypothesis that drugs with similar side effects and ATC codes tend to act on similar targets, respectively. We compared DSESI and DTSI with aforementioned DBSI and found that the performance of the three methods is DTSI > DBSI > DSESI.

Currently, similarity inference methods also have several pitfalls. Their application domains are often limited by lack of similarity data. For example, DSESI and DTSI cannot be used to predict potential DTIs for new chemical entities due to lack of side-effect data or ATC codes. Moreover, all these type of methods did not consider the interaction types and binding affinities.

### Random Walk-Based Methods

Random walk is a classical concept which has been employed in various research areas, such as recommender systems (Liu and Lu, 2010) and prediction of gene-disease associations (Kohler et al., 2008). They can also be used for DTI prediction (Chen et al., 2012).

The most representative example of random walk-based method is Network-based Random Walk with Restart on the Heterogeneous network (NRWRH) (Chen et al., 2012). NRWRH predicts potential DTIs by performing random walk with restart on the heterogeneous network, which is constructed by integrating the known DTI network with chemical similarity of drugs and protein sequence similarity of targets. Based on systematic evaluation, NRWRH outperforms several machine learning-based methods (Chen et al., 2012). After that, NRWRH was improved by employing new types of similarity calculated by extended connectivity fingerprints, 2D pharmacophore fingerprints and ROCS program (Seal et al., 2015).

Mathematically, the descriptions of random walk-based methods are similar to those of the aforementioned NBI series methods. They use matrix multiplication to describe the random-walk and resource-diffusion processes, respectively. Owing to the similarity between these two types of methods, random walk-based methods have similar defects that NBI series methods have, such as cannot predict interaction types and binding affinities.

### Local-Community-Paradigm Methods

The local-community-paradigm (LCP) theory was developed for link prediction in monopartite networks such as brain connectomes and protein interactomes (Cannistraci et al., 2013). Then, this theory was extended to bipartite networks (Daminelli et al., 2015). Recently, five LCP methods, including Cannistraci-Alanis-Ravasi (CAR), Cannistraci-Jaccard (CJC), Cannistraci preferential attachment (CPA), Cannistraci-Adamic-Adar (CAA), and Cannistraci resource allocation (CRA), were applied in prediction of potential DTIs and showed high performance (Duran et al., 2017). Currently, these LCP-based methods only rely on the known DTI network. Hence, they would meet the challenges that NBI has met, namely predicting potential DTIs for new chemical entities and targets outside of the known DTI network, as well as considering interaction types and binding affinities.

### Other Network-Based Methods

In addition, there are some other types of network-based methods. For instance, DASPfind uses simple paths of particular lengths on the heterogeneous network for DTI prediction (Ba-Alawi et al., 2016). Similar to the aforementioned NRWRH (Chen et al., 2012), the heterogeneous network is also constructed by integrating DTI network with drug similarity and target similarity. In performance evaluation, DASPfind outperformed several previously published network-based methods such as NRWRH (Chen et al., 2012) and DT-Hybrid (Alaimo et al., 2013).

## Evaluation Of Network-Based Methods

### Cross Validation

The 10-fold cross validation is an approach commonly used to evaluate the robustness of the models built via network-based methods (Cheng et al., 2012b,d; Wu et al., 2016, 2017, 2018; Fang et al., 2017b). In a 10-fold cross validation process, 10% of the DTIs are randomly extracted from the known DTI network as the test set in turn, while the remnant are used as the training set. Hence, 10 pairs of training set and test set are generated. Using these pairs, several evaluation indicators can be calculated. Generally, for a network-based model, the 10-fold cross validation process will be repeated several (e.g., 10) times to reduce the randomness. Finally, based on the results from all 10-fold cross validation processes, the evaluation indicators can be expressed as mean ± standard deviation (SD) or mean ± standard error of the mean (SEM).

### External Validation

The external validation is an approach commonly used to evaluate the generalization ability of the models built via network-based methods (Cheng et al., 2012d; Wu et al., 2016, 2017; Fang et al., 2017b). Before validation, we need to collect a lot of extra experimentally validated DTIs which do not existed in the known DTI network. Then, the whole known DTI network are used as the training set, while the collected extra DTIs are used as the test set. In external validation, the test set is commonly known as external validation set. Using this pair of training set and test set, evaluation indicators can be calculated.

### Evaluation Indicators

Currently, to our knowledge, there are several types of evaluation indicators. For network-based methods, a popular type of evaluation indicators is from recommender systems (Zhou et al., 2010; Lu et al., 2012), such as precision (*P*), recall (*R*), precision enhancement (*e*_P_), and recall enhancement (*e*_R_). Compared with those widely used in evaluating machine learning models for prediction of ADMET properties (Cheng et al., 2012a, 2013a) and DTIs (Cheng et al., 2012c), such as sensitivity and specificity, the evaluation indicators from recommender systems are more personalized and thus suitable for the network-based models that were derived from recommendation algorithms (Cheng et al., 2012b,d; Wu et al., 2016, 2017, 2018; Fang et al., 2017b). Although several evaluation indicators (e.g., *P* and *R*) have the same name as those used in evaluating machine learning models, their definitions are different. Herein, we would briefly described how to calculate the personalized evaluation indicators using a pair of training set and test set, referred as to our previous studies (Cheng et al., 2012b,d; Wu et al., 2016, 2017, 2018; Fang et al., 2017b).

At first, for the pair of training set and test set, nodes which lost all its edges in the training set are removed from both the training set and the test set. After predicting all potential DTIs using the training set and required additional information [e.g., drug-substructure associations for SDTNBI (Wu et al., 2017) and bSDTNBI (Wu et al., 2016)], evaluation indicators can be calculated by comparing the predicted DTIs with the known DTIs in the test set. In general, drugs without known DTIs in the test set do not participate in calculation to avoid invalid values such as infinite. For each drug *D*_i_ participated in calculation, the newly predicted DTIs of *D*_i_ are sorted by their predictive scores. Then, under a user-given threshold such as *L* = 20, the DTIs ranked in the top-*L* places are considered as positive, whereas the others were considered as negative. By comparing those newly predicted DTIs of *D*_i_ that are considered as positive or negative with the known DTIs of *D*_i_ in the test set, the numbers of true positives *TP*_i_(*L*), false positives *FP*_i_(*L*), true negatives *TN*_i_(*L*), and false negatives *FN*_i_(*L*) were counted. After counting the four numbers for each drug participated in calculation, four evaluation indicators *P, R, e*_P_, and *e*_R_ can be calculated as:

(1) $$P(L)=\frac{1}{\text{M}}\cdot \sum_{\text{i}=1}^{M}\frac{TP_{\text{i}}(L)}{TP_{\text{i}}(L)+FP_{\text{i}}(L)}=\frac{1}{M}\cdot \sum_{\text{i}=1}^{\text{M}}\frac{TP_{\text{i}}(L)}{L}$$

(2) $$R(L)=\frac{1}{\text{M}}\cdot \sum_{\text{i}=1}^{M}\frac{TP_{\text{i}}(L)}{TP_{\text{i}}(L)+FN_{\text{i}}(L)}=\frac{1}{M}\cdot \sum_{\text{i}=1}^{M}\frac{TP_{\text{i}}(L)}{X_{\text{i}}}$$

(3) $$e\text{p}(L)=P(L)\cdot \frac{M\cdot N}{\sum_{\text{i}=1}^{\text{M}}X_{\text{i}}}$$

(4) $$e_{R}(L)=R(L)\cdot \frac{N}{L}$$

In these formulas, *M* and *N* are the number of drugs and targets participated in calculation, respectively, and *X*_i_ is the number of known DTIs of *D*_i_ in the test set.

However, all the four evaluation indicators depend on the threshold *L*, whose value may be hard to choose by users. Hence, an evaluation indicator independent of the *L* value, namely area under receiver operating characteristic curve (AUC), is usually employed (Wu et al., 2016, 2017, 2018; Fang et al., 2017b). The calculation of the AUC value can also be described using the aforementioned *TP*_i_(*L*), *FP*_i_(*L*), *TN*_i_(*L*), *FN*_i_(*L*). For a given *L* value, a true positive rate (TPR) and a false positive rate (FPR) can be calculated as:

(5) $$TPR(L)=\frac{\sum_{\text{i}=1}^{\text{M}}TP_{\text{i}}(L)}{\sum_{\text{i}=1}^{\text{M}}TP_{\text{i}}(L)+\sum_{\text{i}=1}^{\text{M}}FN_{\text{i}}(L)}$$

(6) $$FPR(L)=\frac{\sum_{\text{i}=1}^{\text{M}}FP_{\text{i}}(L)}{\sum_{\text{i}=1}^{\text{M}}FP_{\text{i}}(L)+\sum_{\text{i}=1}^{\text{M}}TN_{\text{i}}(L)}$$

By setting different *L* values, for example, varying *L* from 0 to *N*, a series of TPRs and FPRs can be obtained. Then, a receiver operating characteristic (ROC) curve can be generated by plotting the TPRs against the FPRs. The AUC is the area under the ROC curve.

For a model, higher *P, R, e*_P_, *e*_R_, and AUC values obtained in cross validation or external validation indicate higher performance, generally.

## Applications In Target Prediction

A major application area of the network-based methods for DTI prediction is target prediction. By combining target prediction and experimental validation, we may exploit new uses for approved drugs (Cheng et al., 2012b) and new chemical entities (Wu et al., 2016, 2018), and hence facilitate drug repurposing (also known as drug repositioning) and new drug discovery.

### Target Prediction for Approved Drugs

Drug repurposing usually has lower costs and higher successful rate in contrast to new drug discovery (Ashburn and Thor, 2004; Chong and Sullivan, 2007). Over the past decade, various computational methods were developed for drug repurposing (Vanhaelen et al., 2017). A classical strategy is to predict new indications for old drugs directly. For example, a method named MANTRA predicts therapeutic effects for drugs by indentifying network communities in a drug-drug network constructed by calculating the gene expression profile similarity (Iorio et al., 2010). Another method named PREDICT employs multiple drug similarity and disease similarity for large-scale prediction of drug indications (Gottlieb et al., 2011). Recently, bi-directional random walk was also employed to predict drug-disease associations (Luo et al., 2016).

However, targets were not included in the framework of these methods. It may be difficult to understand molecular mechanisms of the new indications. Network-based methods can solve this pitfall and be fruitfully applied in this area. Via an indirect strategy, we may predict potential targets for approved drugs, and hence discover potential new indications of the drugs. For instance, in a previous study (Cheng et al., 2012b), we performed the NBI method on a global DTI network. Nine and 31 approved drugs predicted to target dipeptidyl peptidase IV (DPP4) and estrogen receptors (ERs) with high predictive scores were purchased for experimental assays, respectively. Among the 40 purchased approved drugs, montelukast on DPP4 as well as diclofenac, simvastatin, ketoconazole, and itraconazole on ERs, were validated by *in vitro* bioassays with IC_50_ or EC_50_ values less than 10 μM (**Table 3**). Furthermore, simvastatin and ketoconazole showed anti-proliferative activities on human MDA-MB-231 breast cancer cell line in MTT assays with IC_50_ values less than 10 μM, suggesting that these antifungal agents may have therapeutic effects on breast cancer.

**Table 3:** Application examples of network-based methods in target prediction.

Compound name	Compound type	Original primary targets	Newly discovered targets	Reference
Montelukast	Approved drug	CYSLTR1	DPP4 (IC_50_ = 9.79 μM)	Cheng et al., 2012b
Diclofenac	Approved drug	PTGS1, PTGS2	ERα (IC_50_ = 7.59 ± 0.10 μM) ERβ (IC_50_ = 2.32 ± 0.06 μM)	Cheng et al., 2012b
Simvastatin	Approved drug	HMGCR	ERβ (IC_50_ = 3.12 ± 0.01 μM)	Cheng et al., 2012b
Ketoconazole	Approved drug	ERG11	ERβ (IC_50_ = 0.79 ± 0.15 μM)	Cheng et al., 2012b
Itraconazole	Approved drug	ERG11	ERα (EC_50_ = 0.20 ± 0.41 μM) ERβ (IC_50_ = 0.28 ± 0.73 μM)	Cheng et al., 2012b
AM966	Experimental drug	LPARs	PTGER4 (IC_50_ = 2.67 μM in calcium flux assay, IC_50_ = 2.31 μM in cAMP assay)	Wu et al., 2018
Ki16425	Experimental drug	LPARs	PTGER4 (IC_50_ = 6.34 μM in calcium flux assay, IC_50_ = 5.72 μM in cAMP assay)	Wu et al., 2018

*The original primary targets of these compounds were collected from DrugBank (Wishart* et al., *2018) and IUPHAR/BPS Guide to PHARMACOLOGY (Harding* et al., *2018). CYSLTR1, cysteinyl leukotriene receptor 1; PTGS1, prostaglandin G/H synthase 1; PTGS2, prostaglandin G/H synthase 2; HMGCR, 3-hydroxy-3-methylglutaryl-coenzyme A reductase; ERG11, lanosterol 14-alpha demethylase; LPARs, lysophosphatidic acid receptors; DPP4, dipeptidyl peptidase 4; ERα, estrogen receptor α; ERβ, estrogen receptor β; PTGER4, prostaglandin E2 receptor EP4 subtype*.

### Target Prediction for New Chemical Entities

For new chemical entities, we would briefly describe two examples of finding new active compounds on nuclear receptors (Wu et al., 2016) and GPCRs (Wu et al., 2018), respectively.

In a previous study (Wu et al., 2016), we screened potential ligands for a nuclear receptor named ERα, which served as a potential target for ERα-positive breast cancer (Nilsson et al., 2011), from the Enamine database^1^ via a strategy combining 2D chemical similarity searching and the bSDTNBI method. From the prediction results, 56 commercially available compounds predicted to target ERα were purchased for *in vitro* assays. 27 of them showed potential activities on ERα with IC_50_ or EC_50_ less than 10 μM, suggesting the high performance of our bSDTNBI method (Wu et al., 2016). These new ERα ligands may provide lead compounds for the targeted therapy of ERα-positive breast cancer.

Recently, to investigate polypharmacology of GPCR ligands, we constructed global network-based models for human GPCRs via the bSDTNBI method and three types of molecular fingerprints (Wu et al., 2018). The global network-based model with the best performance in cross validation was employed to predict potential new GPCR targets for known GPCR ligands. 20 compounds predicted to target a GPCR named prostaglandin E2 receptor EP4 subtype were purchased for *in vitro* assays. Among these purchased compounds, AM966 and Ki16425, two known antagonists for lysophosphatidic acid receptors, showed potential antagonistic activities on EP4 in both calcium flux assay and cAMP assay with IC_50_ values less than 10 μM (**Table 3**), providing potential lead compounds for the therapy of colon cancer, lung cancer, osteoporosis and rheumatoid arthritis (Wu et al., 2018).

## Applications In Elucidation Of Molecular Mechanisms

Another major application area of the network-based methods for DTI prediction is to elucidate potential molecular mechanisms of therapeutic effects or safety problems (e.g., toxicity and side effects), which may facilitate systems pharmacology or systems toxicology.

A commonly used approach of deciphering molecular mechanisms is to construct and analyze drug-gene-disease networks (Cheng et al., 2013c,d; Wu et al., 2016, 2017, 2018; Fang et al., 2017b). Specifically, for a class of drugs or a type of disease of interests, a drug-gene-disease network can be constructed by integrating the known and predicted DTIs with gene-disease associations. The gene-disease associations are usually collected from databases such as CTD (Davis et al., 2017), HuGE Navigator (Yu et al., 2008), Online Mendelian Inheritance in Man (Amberger et al., 2015), and PharmGKB (Hewett et al., 2002). After construction of the drug-gene-disease network, network visualization tools such as Cytoscape (Smoot et al., 2011) can be used to show the network visually. In addition to network visualization, various bioinformatics enrichment tools (Huang et al., 2009), such as gene set enrichment analysis (Subramanian et al., 2005), can be employed to analyze the functions of the genes in the network. Based on the systematic analysis results from different angles as well as previously published data and literature in pharmacology and clinics, we may understand molecular mechanisms of the drugs in the drug-gene-disease network.

In this section, we provided several examples of elucidating molecular mechanisms of therapeutic effects or safety problems for approved drugs (Cheng et al., 2013c; Wu et al., 2016, 2017, 2018), natural products (Fang et al., 2017b) and xenobiotics (Cheng et al., 2013d) via network-based methods.

### Elucidation of Molecular Mechanisms of Therapeutic Effects

Recent studies have shown that the use of NSAIDs is associated with lower risk of cancer (Nan et al., 2015). However, molecular mechanisms of the chemoprevention by NSAIDs are still not well understood. In a previous case study of the SDTNBI method (Wu et al., 2017), we used the SDTNBI method to predict potential DTIs for NSAIDs. A drug-gene-disease network containing 21 NSAIDs and 29 cancer types or subtypes were constructed by integrating the known and predicted DTIs with gene-disease associations. Several newly predicted DTIs were validated by previously reported literature, suggesting the high performance of our SDTNBI method. After performing systematic analysis on the drug-gene-disease network using previously published pharmacological experiments and co-crystal structure data, we found that NSAIDs may exert anticancer effect by inhibiting their targets associated with cancer, such as prostaglandin G/H synthase 2 (PTGS2, also known as cyclooxygenase-2), aldo-keto reductase family 1 member C3 (AKR1C3), carbonic anhydrase 9 (CA9), carbonic anhydrase 12 (CA12) and cyclin-dependent kinase 2 (CDK2).

Subsequently, in a case study of the bSDTNBI method (Wu et al., 2016), we investigated molecular mechanism of anticancer effects of approved drugs in a larger scale. After predicting potential DTIs for approved drugs via the bSDTNBI method, a global drug-gene-disease network containing 666 approved drugs and 15 cancer types or subtypes were built by integrating the known and predicted DTIs with the gene-disease associations. Based on systematic analysis, we demonstrated that tricyclic anti-depressant drugs and anti-diabetic drugs may exert anticancer effects by targeting serotonin receptors and cancer cell metabolism, respectively. These two case studies showed the practical application of network-based methods in elucidating therapeutic effects of approved drugs.

Besides approved drugs, network-based methods can also be used to elucidate the therapeutic effects of natural products. For example, in a recent study (Fang et al., 2017b), we utilized the bSDTNBI method to build global network-based models for natural compounds from traditional chinese medicine (TCM) databases. The best bSDTNBI model in cross validation was used to predict potential DTIs for natural products. Then, potential anticancer indications of the natural products were further predicted via gene-disease associations for cancer collected from public available databases and a statistical approach based on permutation test (Fang et al., 2017b). Taking three natural products, namely kaempferol, resveratrol, and genistein, as examples, drug-gene-disease networks were constructed. After performing systematic analysis using the networks and previously published evidence, we found that the three natural products may exert anticancer effects by inhibiting different cancer-associated proteins and pathways. Moreover, the similar workflow was also used to investigate molecular mechanism of anti-aging effects of natural products (Fang et al., 2017a).

### Elucidation of Molecular Mechanisms of Safety Problems

In addition to therapeutic effects, network-based models can also be applied in elucidating molecular mechanisms of safety problems, such as side effects (Cheng et al., 2013c) and toxicity (Cheng et al., 2013d).

For side effects, in a recent study (Wu et al., 2018), the bSDTNBI method was used to predict potential targets for GPCR drugs. Then, drug-gene-disease networks were constructed for two example GPCR drugs named clemastine and dobutamine, by integrating the known and predicted DTIs as well as side-effect data from MetaADEDB (Cheng et al., 2013b) and Lounkine’s study (Lounkine et al., 2012). Via systematic analysis on the networks, we identified that the cardiovascular complications of GPCR drugs were associated with their off-target effects on α-adrenergic receptor and muscarinic acetylcholine receptors.

For toxicity, in a previous study (Cheng et al., 2013d), we proposed a computational systems toxicology framework based on the NBI method, named predictive toxicogenomics-derived models (PTDMs), to help understand how the xenobiotics (e.g., drugs, industrial chemicals and pesticides) influence human health and the environment. At first, three networks were constructed for chemical-gene interactions (CGIs), chemical-disease associations (CDAs) and gene-disease associations (GDAs). Herein, different from the above studies, the CGIs included both direct chemical-protein interactions and indirect chemical-gene associations. Then, new potential CGIs, CDAs, and GDAs were prioritized via performing the NBI method on the three networks, respectively. Based on the known and predicted chemical-gene-disease association data, we systematically investigate the toxicological mechanisms of an endocrine disrupter named bisphenol A (BPA). Some predicted associations for BPA were in agreement with previously published data, suggesting the potential application of network-based methods in elucidating the toxicological mechanisms of xenobiotics.

All these examples of elucidating molecular mechanism of therapeutic effects or safety problems illustrate the potential applications of network-based methods in systems pharmacology and systems toxicology. However, to date, most of the studies are just pure computational studies. Further biological assays and clinical studies are needed to validate the predictive results in the future.

## Summary And Perspectives

Since the new century, as the rapid development of systems biology and network pharmacology, various computational methods were proposed for DTI prediction with high efficiency and low costs (Zheng et al., 2013; Chen et al., 2016; Lavecchia and Cerchia, 2016). Among these methods, network-based methods have shown obvious advantages (Cheng et al., 2012b,d; Wu et al., 2016, 2017). As mentioned above, this category of methods relies on neither 3D structures of targets nor negative samples, which can cover much larger target space. Although network-based methods only perform simple mathematical operations such as matrix multiplication in prediction, high performance has shown not only in theory but also in potential applications, including target prediction (Cheng et al., 2012b; Wu et al., 2016, 2018) as well as elucidation of molecular mechanisms of both therapeutic effects (Wu et al., 2016, 2017; Fang et al., 2017b) and safety problems (Cheng et al., 2013c,d; Wu et al., 2018).

Despite the success of currently available network-based methods, there are still several pitfalls. First, the application domain of these methods still needs to be extended. Although several recently proposed network-based methods can be used to predict potential DTIs for both approved drugs and new chemical entities (Wu et al., 2016, 2017), they cannot predict potential DTIs for those targets without known ligands. Second, network-based methods are still non-quantitative. They only provide a predictive score for each potential DTI, where a higher score means a higher probability of occurrence (Cheng et al., 2012b,d; Wu et al., 2016, 2017). The binding affinities of the predicted DTIs are unknown. Moreover, the interaction type is not considered yet. In the real world, there are different types of DTIs. For example, receptors have agonists, antagonists and inverse antagonists, while enzymes have activators and inhibitors. However, to our knowledge, no network-based methods have considered the interaction type yet—DTIs were simply seen as indirect edges without any additional attributes. By contrast, efforts have been made in other types of computational methods in these years. In the aspect of quantitative prediction, as described in the INTRODUCTION section, many structure-based methods have the ability to predict binding affinities of DTIs (Aldeghi et al., 2017; Liu et al., 2017). In the aspect of prediction of interaction types, Wang and Zeng (2013) have proposed the first machine learning-based method to predict the interaction type. To avoid losing competitiveness, it is urgently needed to develop novel network-based methods.

In our views, there are several possible strategies to further improve the network-based methods for DTI prediction. At first, we can try to introduce new link prediction algorithms into our research area, such as hierarchical structure (Clauset et al., 2008), stochastic block (Guimera and Sales-Pardo, 2009), and likelihood analysis (Pan et al., 2016). Secondly, we can integrate multi-scale biomedical data, including drug-side effect associations (Tatonetti et al., 2012; Kuhn et al., 2016), drug-indication associations (Brown and Patel, 2017), drug-induced gene expression profiles (Lamb et al., 2006; Subramanian et al., 2017), protein-protein interactions (Li et al., 2017), ADMET properties (Cheng et al., 2012a), clinical data (Zarin et al., 2011), etc. In addition, we can learn something from structure-based methods and make full use of 3D structures of targets. Although many targets do not have 3D structures yet, the already resolved 3D structures are valuable information. For example, as a simplest way, we can use docking scores to improve the predictive scores of network-based methods via a consensus approach. These may help us move toward structural systems pharmacology (Xie et al., 2014).

In summary, although the network-based for DTI prediction still have limitations, they provide alternative tools for studies in drug repurposing, new drug discovery, systems pharmacology and systems toxicology. We hope they would play a greater role in the future.

## Author Contributions

YT conceived and directed the project. YT and ZW designed the review and wrote the manuscript. WL and GL provided useful advice. All authors read and approved the final version of the manuscript.

## Conflict of Interest Statement

The authors declare that the research was conducted in the absence of any commercial or financial relationships that could be construed as a potential conflict of interest.

## Footnotes

1 http://www.enamine.net/
